# SARS‐CoV‐2 infection complicated with cold agglutinin disease and myositis

**DOI:** 10.1002/ccr3.3981

**Published:** 2021-03-16

**Authors:** Fadi Aldaghlawi, Alaa Shammah, Ebenezer Kio

**Affiliations:** ^1^ Department of Medicine Goshen Health Hospital Goshen IN USA; ^2^ Department of Medicine Section of hematology & oncology Goshen Center for Cancer Care Goshen IN USA

**Keywords:** anemia, cold agglutinin, hemolytic, myositis, SARS‐CoV‐2

## Abstract

This case emphasizes the importance of prompt comprehensive investigation of anemia and myositis in patients infected with SARS‐CoV‐2 and early recognition of uncommon complications that can be associated with SARS‐CoV‐2 infection.

## Graphical abstract

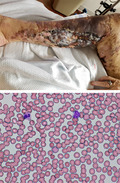

## 1 INTRODUCTION

Respiratory symptoms are the main manifestation of SARS‐CoV‐2 infection. Extrapulmonary complications are increasingly reported. Myositis has been reported as a complication of viral infections.^1^ It has also been reported in association with SARS‐CoV‐2.^2^ While several immune‐related complications have been linked to this infection, other than coagulation abnormalities very little information is available describing hematologic complications.^3^ Herein, we report the case of a female patient who developed cold agglutinin disease and myositis as a complication of acute SARS‐CoV‐2.

## 2 CASE REPORT

A 69‐year‐old female with a history of stage IV chronic lymphocytic leukemia on tirabrutinib presented to the emergency room with low‐grade fever, arthralgias, myalgias, and nonproductive cough for 2 days. She was hemodynamically stable with normal oxygen saturation on room air. Nasopharyngeal swab for reverse transcriptase‐polymerase chain reaction (RT‐PCR) test obtained for SARS‐CoV‐2. She was discharged home on empiric levofloxacin 750 mg daily, and tirabrutinib was discontinued. Two days later, a positive test for RT‐PCR for SARS‐CoV‐2 was returned.

On day 9 of onset of symptoms, she was admitted with worsening dyspnea, anorexia, and fatigue. Her vital signs were as follows: temperature of 100.3 Fahrenheit, pulse 99 bpm, respiratory rate 24 breaths per min, blood pressure 128/76 mm Hg, and oxygen saturation SpO2 89% on ambient air. She was continued on levofloxacin 750 mg daily and started on tapered dose dexamethasone of 6 mg daily. Then, she was discharged home on supplemental oxygen of 2 liters nasal cannula on day 16. Quick COVID‐19 Severity Index score was 12 which indicated a high risk of critical illness.

**FIGURE 1:**
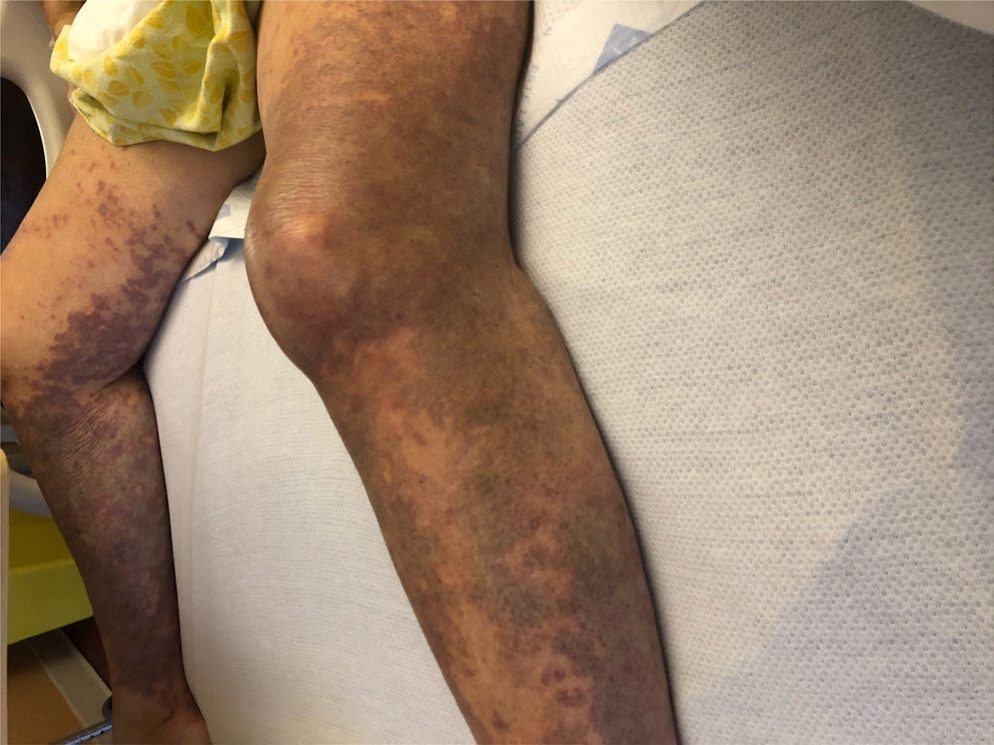
The photograph documents the cutaneous lesions on the bilateral lower extremities on day 18 of onset of SARS‐CoV‐2 symptoms

**FIGURE 2:**
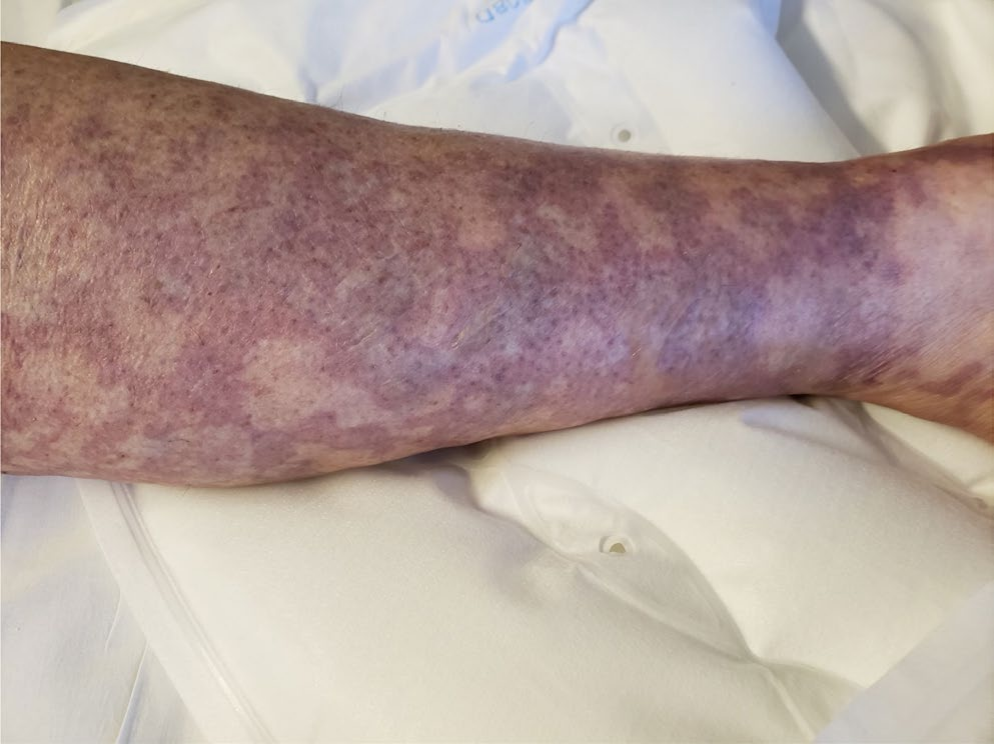
The photograph documents the cutaneous lesions on the right lower extremities on day 18 of onset of SARS‐CoV‐2 symptoms

On Day 18, she was readmitted with severe intractable pain in her bilateral lower extremities with subjective pelvic girdle weakness. Physical examination revealed petechial lesions and reticulated vascular violaceous cutaneous lesions on the anterior chest wall, tip of the nose, lips, bilateral lower extremities sparing anterior abdominal wall and truncal areas (Figures 1,2). Severe tenderness and hyperalgesia elicited by palpation in the bilateral calves and quadriceps musculature with acrocyanosis affecting the tip of fingers, nose, and malar prominence. Strong symmetric distal pulses were palpated. Motor examination revealed symmetric grade 4/5 power in upper extremities and grade 2/5 in power lower extremities.

Laboratory examination results were as follows: creatine phosphokinase 2713 µ/L, lactate dehydrogenase 1348 µ/L, haptoglobin 196 mg/dL, hemoglobin 11.7 gm/dL, platelets 75 k/mm^3^, aspartate aminotransferase 96 µ/L, alanine aminotransferase 72 µ/L, creatinine 0.6 mg/dL, prothrombin 12.3 seconds, partial thromboplastin time 32.5 seconds, fibrinogen 599 mg/dL, IGG 333 mg/dL, immunoglobulin M 26 mg/dL, immunoglobulin A 83 mg/dL, hepatitis B and C viral serologies were negative for acute infection.

**FIGURE 3:**
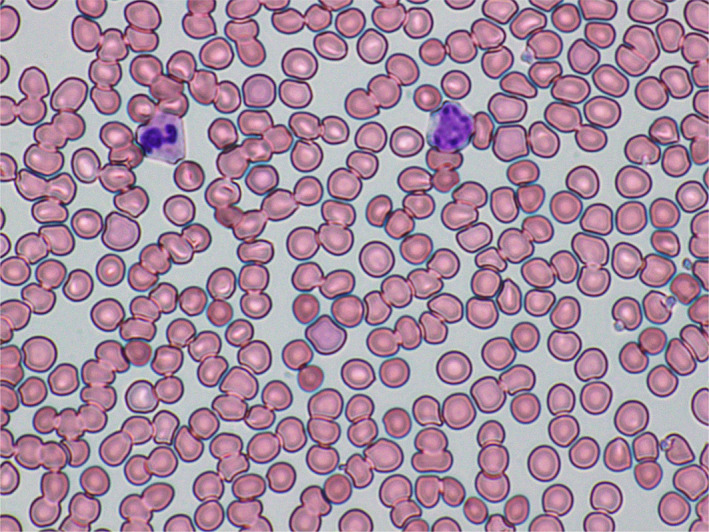
Microscopic photograph of the blood peripheral smear showing marked agglutination on day 18 of onset of SARS‐CoV‐2 symptoms

**FIGURE 4:**
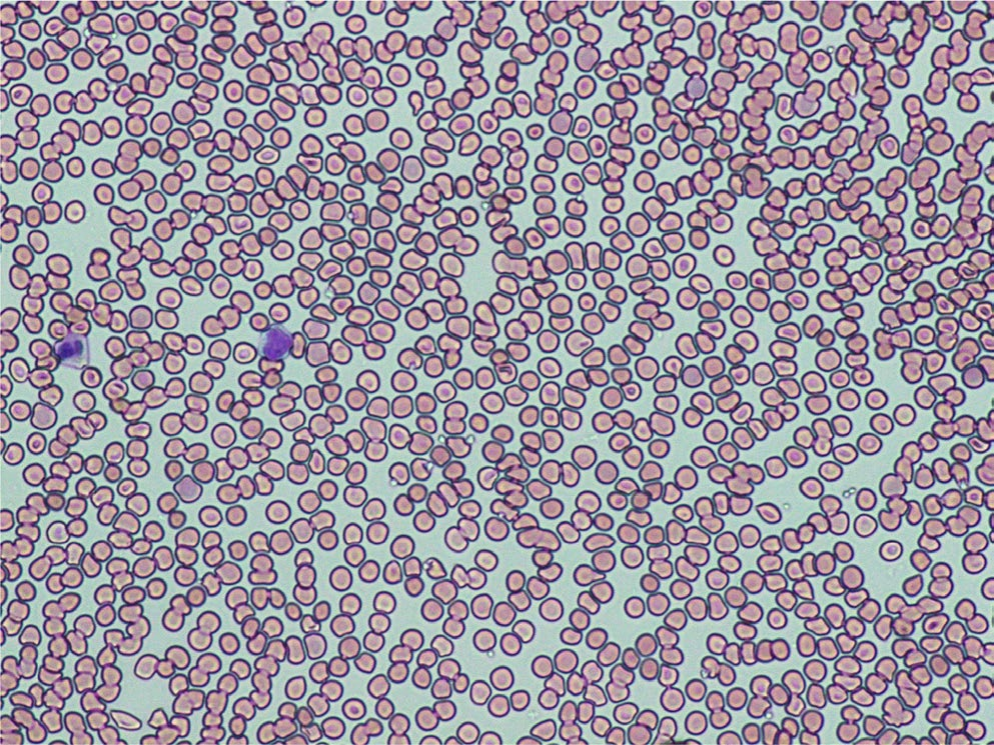
Microscopic photograph of the blood peripheral smear showing marked agglutination on day 18 of onset of SARS‐CoV‐2 symptoms

Peripheral blood smear revealed marked agglutination of red blood cells and a cold agglutinin with thermal amplitude of 30°C was identified with complement C3B and C4 identified on red blood cells (Figures 3,4). Peripheral blood smear showed normal‐sized platelets, normal‐appearing neutrophils with toxic granulation, atypical lymphocytes somewhat eccentric nuclei, occasional nucleated red blood cells, and mild polychromasia with smudge cells.

**FIGURE 5:**
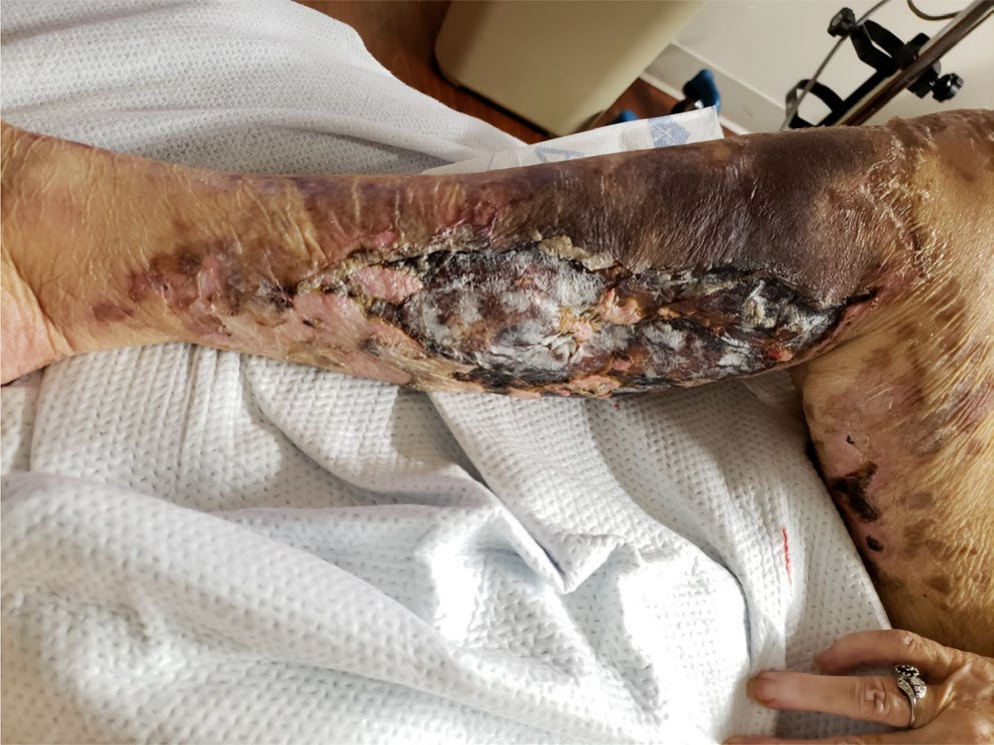
The photograph documents the increased mottling and worsening cutaneous lesions with new bulla formation and open wounds in the right lower extremities on day 20 of onset of SARS‐CoV‐2 symptoms

On Day 20, physical examination was notable for new bulla formation in the lower extremities and increased mottling of upper extremities (Figure 5). Laboratory studies showed worsening thrombocytopenia (platelets 11 k/mm^3^) and anemia (hemoglobin 6.7 gm/dL). Treatment with prednisone 1 mg/kg daily for a total of 4 weeks was continued. Rituximab 375 mg/m^2^ weekly × 4 doses was initiated for cold agglutinin hemolytic anemia, and intravenous immunoglobulin infusion was administered at 1 g/kg daily × 2 doses on day 21 to address possible immune‐related thrombocytopenia.

On Day 26, the patient was discharged to a rehabilitation facility with a normal creatinine phosphokinase of <20 U/L and stable hemoglobin of 10.5 gm/dL.

Five months later, cutaneous ulcers completely resolved following local wound and supportive care without skin grafts.

## 3 DISCUSSION

Cold agglutinin disease is an immune‐mediated hemolytic anemia caused by immunoglobulin M‐complement mediated lysis of red blood cells. Immunoglobulin M attaches to red blood cells in cooler parts of the body. There are two kinds of cold agglutinin disease: Primary that causes red blood cell agglutination and extravascular hemolysis without an underlying disorder and secondary when cold agglutinins develop in the setting of an underlying condition such as a viral infection, autoimmune disorder, or lymphoid malignancy.^4^

Myositis is an inflammation of a muscle, which can present with pain, tenderness, swelling, and/or weakness.^5^ Influenza A and B and enterovirus are among the common viral etiologies that have been linked to myositis.^6^ The mechanism of muscle necrosis in acute viral myositis remains unclear. However, probable mechanisms are an invasion of muscle tissue by the viral agent, myotoxic cytokines release in viral infection, or immunologic processes induced by a viral infection, which could result in muscle damage.^7^ Also, rhabdomyolysis has been reported in association with SARS‐Cov‐2.^2^

The pathophysiology underlying severe SARS‐CoV‐2 remains unclear and poorly understood, but there has been increasing evidence proposing hyperinflammatory syndrome causing severe cytokines release associated with poor outcome and increased severity of the disease.^8^ To our knowledge, simultaneous myositis and cold agglutinin disease associated with SARS‐CoV‐2 have not been previously reported, while generalized body aches, fatigue, and anemia are a common presentation in patients with SARS‐CoV‐2. This case report underscores the importance of keeping a broad differential diagnosis to early recognize uncommon complications that can be associated with SARS‐CoV‐2.

## 4 CONCLUSION

This case emphasizes the importance of prompt comprehensive investigation of anemia and myositis in patients infected with SARS‐CoV‐2. More data are required to ascertain the relation between hemolytic anemia and myositis with SARS‐CoV‐2 infections especially in the setting of underlying malignancy. Further data needed to direct immunosuppression treatment in patients with hematologic complications associated with SARS‐CoV‐2.

## CONFLICT OF INTEREST

None declared.

## AUTHOR CONTRIBUTIONS

FA: performed a literature search and drafted the manuscript. AS: drafted portions of the manuscript and provided Figures 1 and 2. EK: provided revisions for intellectual content and provided pathology slides and Figures 3, 4, 5.

## ETHICS STATEMENT

Written informed consent was obtained from the patient.

## Data Availability

No data are associated with this article.
